# Suppression of LPS-induced inflammatory responses by the hydroxyl groups of dexamethasone

**DOI:** 10.18632/oncotarget.17683

**Published:** 2017-05-08

**Authors:** Ting-Yun Chuang, An-Jie Cheng, I-Ting Chen, Tien-Yun Lan, I-Hsuan Huang, Chung-Wai Shiau, Chia-Lin Hsu, Ya-Wen Liu, Zee-Fen Chang, Ping-Hui Tseng, Jean-Cheng Kuo

**Affiliations:** ^1^ Institute of Biochemistry and Molecular Biology, National Yang-Ming University, Taipei 11221, Taiwan; ^2^ Institute of Biopharmaceutical Sciences, National Yang-Ming University, Taipei 11221, Taiwan; ^3^ Institute of Microbiology and Immunology, National Yang-Ming University, Taipei 11221, Taiwan; ^4^ Institute of Molecular Medicine, College of Medicine, National Taiwan University, Taipei 10002, Taiwan; ^5^ Biophotonics & Molecular Imaging Research Center, National Yang-Ming University, Taipei 11221, Taiwan; ^6^ Proteomics Research Center, National Yang-Ming University, Taipei 11221, Taiwan

**Keywords:** TNF-α secretion, dexamethasone, innate immunity, p38 MAPK signaling, tumor necrosis factor-α (TNF-α)-converting enzyme

## Abstract

The innate immune response is a central process that is activated during pathogenic infection in order to maintain physiological homeostasis. It is well known that dexamethasone (Dex), a synthetic glucocorticoid, is a potent immunosuppressant that inhibits the cytokine production induced by bacterial lipopolysaccharides (LPS). Nevertheless, the extent to which the functional groups of Dex control the excessive activation of inflammatory reactions remains unknown. Furthermore, importantly, the role of Dex in the innate immune response remains unclear. Here we explore the mechanism of LPS-induced TNF-α secretion and reveal p38 MAPK signaling as a target of Dex that is involved in control of tumor necrosis factor-α (TNF-α)-converting enzyme (TACE) activity; that later mediates the shedding of TNF-α that allows its secretion. We further demonstrate that the 11-hydroxyl and 21-hydroxyl groups of Dex are the main groups that are involved in reducing LPS-induced TNF-α secretion by activated macrophages. Blockage of the hydroxyl groups of Dex inhibits immunosuppressant effect of Dex during LPS-induced TNF-α secretion and mouse mortality. Our findings demonstrate Dex signaling is involved in the control of innate immunity.

## INTRODUCTION

Activation of the innate immune system maintains physiological homeostasis when a host has a pathogenic infection, trauma or irradiation [1–3]. The inflammatory response includes a temporal induction of cytokines and various other specific signaling molecules produced by macrophages that restrict ongoing infection [2]. Excessive activation, however, contributes to a number of common human disease states, such as septic shock, asthma, arthritis, and atherosclerosis, and does so by attracting more and more macrophages that then secrete an excess of pro-inflammatory cytokines and chemokines, which results in tissue damage [4–6]. Members of the membrane-associated metalloproteinases have emerged as important modulators of innate immunity via their effects on ectodomain shedding, a type of posttranslational modification; this results in increased secretion of cytokines by activated macrophages [7–12].

The ADAM (a disintegrin and metalloproteinase) family of transmembrane metalloproteinases controls the ectodomain shedding of various substrates [13–15]. The ADAM class of proteases consists of a prodomain, a catalytic domain, a disintegrin domain, a cysteine-rich region, a transmembrane domain, and a cytoplasmic tail. ADAM17, also known as tumor necrosis factor-α(TNF-α)-converting enzyme (TACE) [16, 17], mediates the shedding of inflammatory cytokines and cytokine receptors and thus plays a central role in inflammation [14, 15]. Upon pro-inflammatory stimulation, the activity of TACE is increased by p38 MAPK signaling. Activation of p38 MAPK phosphorylates TACE at Thr735 [18], and the protein is then released from its interaction with tissue inhibitor of metalloproteinase-3 (TIMP3), resulting in its presentation as a monomer at the cell surface [19]. In the absence of p38 MAPK signaling, the activity of TACE is inhibited by the formation of dimers and its interaction with TIMP3 [20, 21]. Thus, TACE activation makes an important contribution to the secretion of pro-inflammatory cytokines such as tumor necrosis factor-α (TNF-α); this controls the innate inflammatory reaction produced by activated macrophages via p38 MAPK signaling.

Glucocorticoids have been well-documented as immunosuppressants and have the ability to inhibit cytokine responses in activated macrophages [22] through transcription [23–26], mRNA stability [25, 27], translation [28, 29] and/or post-translational processing [28]. Glucocorticoids often act through a glucocorticoid receptor in order to exert their anti-inflammatory and cytokine-inhibiting effects [30, 31]. Dexamethasone (Dex), a synthetic glucocorticoid, has been shown to have pleiotropic activity in that it is able to inhibit bacterial lipopolysaccharide (LPS)-induced cytokine production of TNF-α and IL-1β [32, 33], thereby modulating innate immunity in activated macrophages. Dex also impairs LPS-induced activation of p38 MAPK [31], which implies that Dex may inactivate TACE and suppress TNF-α secretion through an inhibition of p38 MAPK signaling. Notwithstanding the above, importantly, the functional groups on Dex remain unknown at present.

Here, we examined whether Dex is able to modulate TACE activity and control LPS-induced innate immunity, and, if it is able to, how it occurs. We used macrophages (RAW264.7 cells) as the model system, since it has been well documented that these cells are able to release pro-inflammatory cytokines such as TNF-α in response to LPS [34]. We found that Dex blocked release of LPS-induced TNF-α, but did not affect the transcriptional and translational expression of induced TNF-α in activated macrophages stimulated by LPS over a 2 hour time periods. Dex is able to bring about the membrane accumulation of induced TNF-α by suppressing p38 MAPK-mediated TACE activation. By examining the effects of Dex on innate immunity, we were also able to determine that the 11-hydroxyl and 21-hydroxyl groups of Dex serve as the molecule's functional groups on activated macrophages, which is required for repression of p38 MAPK signaling, for reducing TACE activity and for inhibiting TNF-α release, all of which help to control the mortality of mice in response to LPS treatment.

## RESULTS

### Suppression of LPS-induced TNF-α secretion by dexamethasone

**Figure 1:**
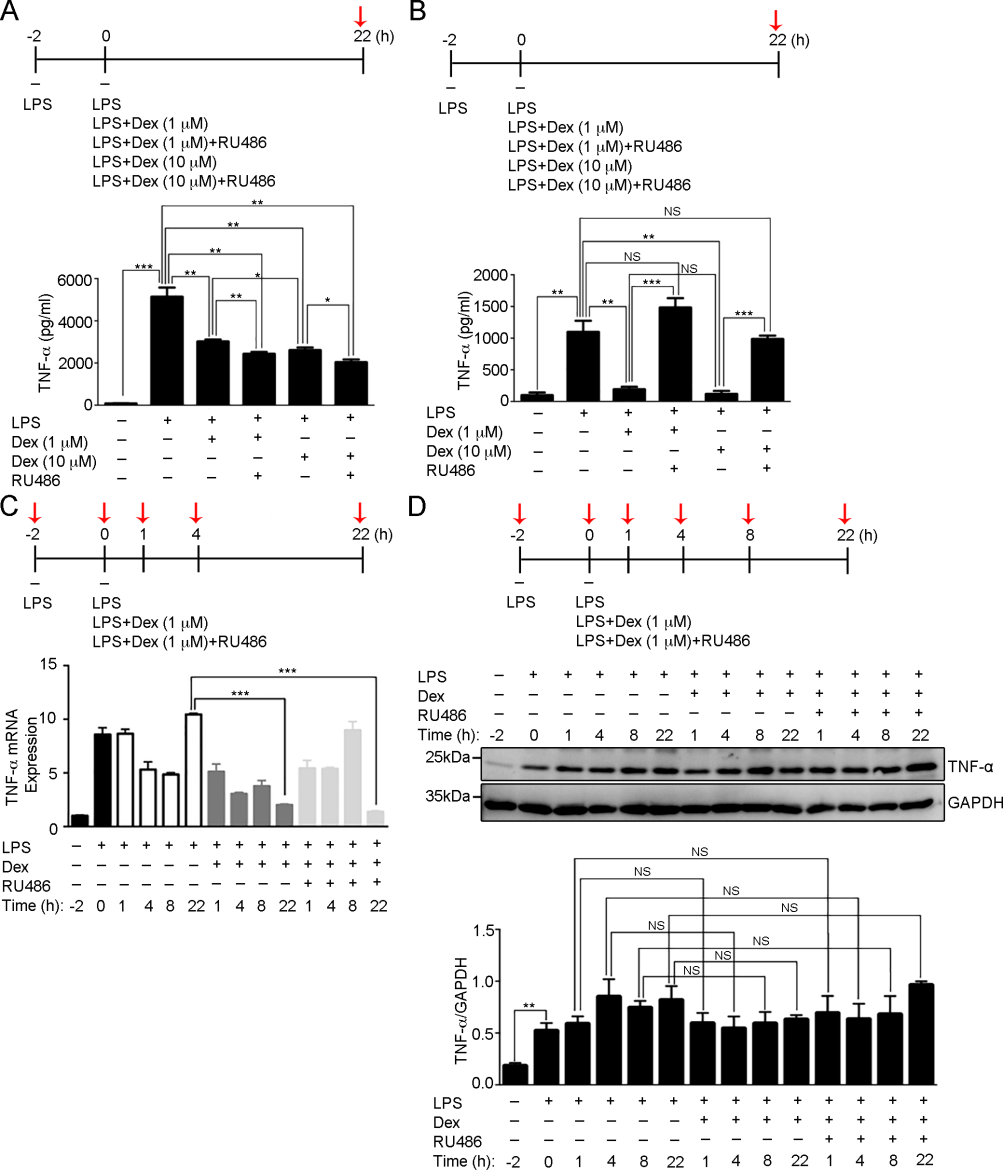
Dexamethasone inhibits LPS-induced TNF-α secretion in activated macrophages **(A)** The concentration of secreted TNF-α was determined in the medium of RAW264.7 cell culture after the indicated treatments at the time point highlighted with red arrow. The graph represents the mean ± s.e.m. (n = 3 independent experiments). *p <0.05; **p <0.01; ***p < 0.001. **(B)** The concentration of secreted TNF-α was determined in the medium of BMDMs culture after the indicated treatments at the time point highlighted with red arrow. The graph represents the mean ± s.e.m. (n = 3 independent experiments). **p <0.01; ***p < 0.001; NS, no significance. **(C)** Fold of TNF-α mRNA expression was determined in RAW264.7 cells after the indicated treatments at the time points highlighted with red arrows relative to the first time point (−2 h). The graph represents the mean ± s.e.m. (n = 3 independent experiments). ***p < 0.001. **(D)** Cell lysates from RAW264.7 cells after the indicated treatments at the time points highlighted with red arrows were analyzed by Western blotting using antibodies against TNF-α and GAPDH. Bottom: the fold expression of TNF-α normalized against GAPDH was determined by Western blotting (n = 3 independent experiments; data are mean ± s.e.m.). **p <0.01; NS, no significance.

To test the hypothesis that the innate immune responses of macrophages are able to be limited by glucocorticoids acting through a glucocorticoid receptor [31], we first examined the effects of Dex on LPS-induced TNF-α secretion in RAW cells and bone marrow-derived macrophages (BMDMs). After 2 hours of LPS treatment (0.1μg/ml) to activate their inflammatory responses, the activated macrophages were treated continuously with either LPS only or with LPS accompanied by Dex (1μM), Dex (1μM)+RU486 (glucocorticoid receptor antagonist), Dex (10μM) or Dex (10μM)+RU486 for 22 hours. It was found that the Dex treatment significantly suppressed LPS-induced TNF-α secretion using either RAW cells or BMDMs (Figure 1A and 1B). However, treatment with RU486 only appeared to have an inhibitory effect on Dex using BMDMs and not when RAW cells were used. This reveals that there seem to be different Dex mechanisms in macrophages differentiated from bone marrow progenitor cells and macrophages differentiated from circulating blood monocytes (Figure 1A and 1B). The results in Figure 1A imply that there is a glucocorticoid receptor-independent mechanism whereby Dex suppresses LPS-induced TNF-α secretion in activated RAW cells.

We next examined the effects of Dex on LPS-induced TNF-α production. Real-time q-PCR revealed that TNF-α mRNA induction occurred as early as 2 hours after LPS stimulation and then subsequently decreased (Figure 1C). Later, a second phase of TNF-α mRNA induction was observed at 22 hours of LPS treatment. To study the effect of Dex on TNF-α mRNA induction, activated RAW cells were continuously treated with LPS+Dex or LPS+Dex+RU486. The results showed that the early phase of induced TNF-α mRNA production was detected at a similar level with that in the LPS-treated cells, thus Dex treatment did not suppress the induction of LPS-induced TNF-α mRNA. However, the second phase of TNF-α mRNA production was very low in the activated RAW cells treated with LPS+Dex or LPS+Dex+RU486 (Figure 1C), which supports existing evidence that autocrine TNF-α activates cell surface TNF-α receptor signaling in order to induce this second phase of TNF-α production [35, 36]. To determine the effect of Dex on the cell surface expression of TNF-α receptor, flow cytometric analysis was performed. This showed that Dex did not change the expression level of LPS-induced TNF receptor 1 on the cell surface (Supplementary Figure 1). These findings indicate that the action of Dex on LPS-activated macrophages does not influence the level of induced TNF-α and cell surface TNF-α receptor, but does suppress TNF-α secretion. Furthermore, we found the level of TNF-α protein induced by LPS was not reduced by Dex treatment until the 22-hour time point (Figure 1D), which implies there is accumulation of TNF-α inside of the cell during Dex treatment.

### Membrane accumulation of TNF-α by dexamethasone

**Figure 2:**
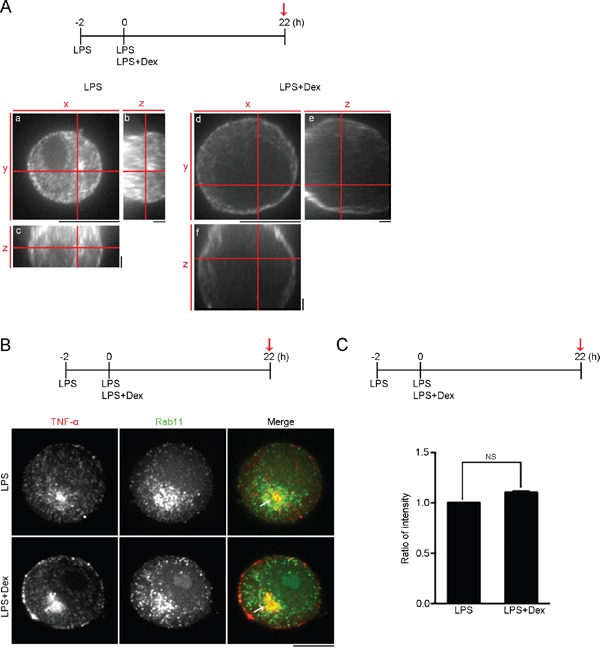
Dexamethasone causes membrane accumulation of TNF-α **(A)** High magnification orthogonal projections of TNF-α staining. Confocal images with orthogonal views demonstrate cellular distribution of TNF-α in RAW264.7 cells treated with 0.1 μg/ml LPS only or 0.1 μg/ml LPS + 1 μM Dex at the time point highlighted with red arrow. (a and d) High magnification XY-average projections of TNF-α. (b and e) High magnification YZ-average projections of TNF-α. (c and f) High magnification XZ-average projections of TNF-α. Scale bars: a and d: 10 μm; b, c, e and f: 2 μm. **(B)** Confocal images of TNF-α and Rab11 double staining of RAW264.7 cells after the indicated treatments at the time point highlighted with red arrow. Bar, 10 μm. **(C)** Ratio of average density of Rab11 within TNF-α-localized regions of the cells treated after the indicated treatments at the time point highlighted with red arrow. (n = 4 cells; data are mean ± s.e.m.). NS, no significance.

To examine the effect of Dex on the cellular distribution of TNF-α in activated macrophages, activated RAW cells (LPS stimulation for 2h) were continuously treated with LPS only or LPS accompanied by Dex for 22 hours, and then immunolabelled using antibodies against TNF-α. Confocal images with orthogonal views were reconstituted from the stack of two-dimensional images and these revealed that there was enrichment of cellular TNF-α at the peripheral regions of the cells treated with LPS and Dex, and this contrasted with the diffused pattern of TNF-α present in LPS-stimulated cells (Figure 2A). Previous evidence has indicated that the TNF-α trafficking machinery uses Rab11 GTPases for the docking and fusion of vesicles at the target membranes [37]. To confirm the effect of Dex on the transportation of synthesized TNF-α, we tracked the cellular distribution of TNF-α and Rab11 within activated RAW cells (LPS stimulation for 2h), within activated RAW cells continuously treated with LPS only for 22 hours or within activated RAW cells continuously treated LPS accompanied with Dex for 22 hours, using immunofluorescence staining. The confocal images revealed that TNF-α co-localized with Rab11 in the activated RAW cells stimulated with either LPS only or LPS+Dex (Figure 2B). We quantified the ratio of fluorescence density of Rab11 in the TNF-α-localized regions and this indicated that Dex did not decrease the Rab11 density to TNF-α signals, within LPS-treated cells (Figure 2C), indicating that Rab11-bound TNF-α-containing vesicles within activated macrophages are not disrupted by Dex treatment. These finding thus suggest that Dex probably suppresses the release of TNF-α from activated macrophages, since TNF-α transportation is not influenced.

### Suppression of TACE activity by dexamethasone via the p38α MAPK pathway

**Figure 3:**
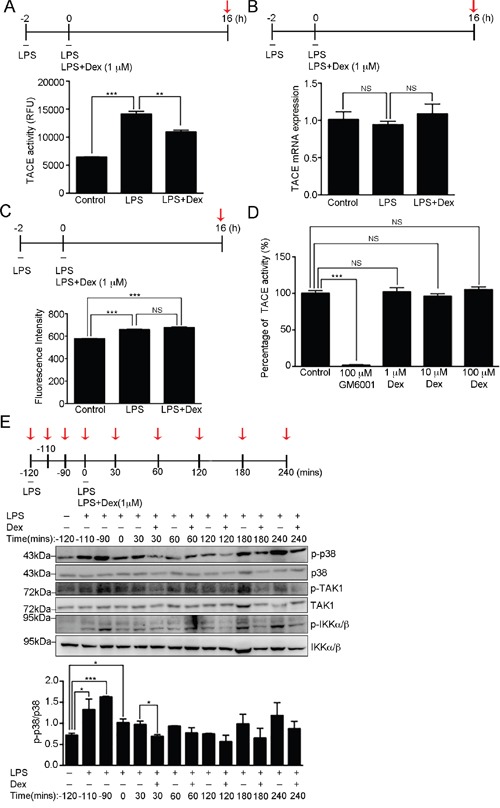
Dexamethasone inhibits TACE activity **(A)** The proteolytic activity of TACE from RAW264.7 cells after the indicated treatments at the time point highlighted with red arrow. The graph represents the mean ± s.e.m. (n = 3 independent experiments). **p <0.01; ***p < 0.001. **(B)** TACE mRNA expression was determined in the RAW264.7 cells after the indicated treatments at the time point highlighted with red arrow relative to cyclophilin A (CPH) mRNA expression. The graph represents the mean ± s.e.m. (n = 3 independent experiments). NS, no significance. **(C)** Analysis of TACE expression on cell surface. RAW264.7 cells with the indicated treatments were incubated with TACE antibody, which was followed by labelling with Cy5-conjugated secondary antibody. Fluorescence intensity was detected using flow cytometry and analyzed by FlowJo software. The graph represents the mean fluorescence intensity ± s.e.m. (n = 3 independent experiments). ***p < 0.001; NS, no significance. **(D)** The percentage of *in vitro* proteolytic activity of recombinant TACE proteins treated with buffer alone (control), 0.1 μM GM6001 (well-known TACE inhibitor), 1 μM Dex, 10 μM Dex or 100 μM Dex. The graph represents the mean ± s.e.m. (n = 3 independent experiments). ***p < 0.001; NS, no significance. **(E)** Cell lysates from RAW264.7 cells with the indicated treatments at the time points highlighted with red arrows were analyzed by Western blotting using antibodies against p38 MAPK, p-p38 [phospho-p38 MAPK (Thr180/Tyr182)], TAK1, p-TAK1 [phospho-TAK1 (Thr184/187)], IKKα/β and p-IKKα/β [phospho-IKKα (Thr176)/IKKβ (Thr177)]. Bottom: fold expression of p-p38 normalized against p38 was determined by Western blotting (n = 3 independent experiments; data are mean ± s.e.m.). *p <0.05; ***p <0.001.

TACE has been shown to promote TNF-α secretion via the efficient shedding of the preform of TNF-α (pro-TNF-α) into the mature soluble form that is released, which results in increased levels of TNF-α in the culture medium [16, 38]. We next examined whether there is Dex suppressed LPS-induced cellular TACE activation. Activated RAW cells (LPS stimulation for 2h) were continuously treated with LPS only or LPS accompanied with Dex for 16 hours, and their cellular TACE activity analyzed. It was found that LPS treatment significantly enhanced TACE activation and this was suppressed when the activated RAW cells were treated with Dex (Figure 3A). To examine whether TACE mRNA expression was suppressed by Dex treatment, real-time q-PCR was performed and this revealed a similar level of TACE mRNA expression at the 16 hour-time point when LPS only or LPS accompanied with Dex cells were compared (Figure 3B). We further examined whether Dex influences the expression level of TACE on the cell surface. RAW cells with the indicated stimuli were stained with antibody against TACE and analyzed by flow cytometry. Figure 3C showed that the fluorescence level of TACE on the cell surface was significantly induced by LPS stimulation, but not inhibited by Dex treatment. Thus, Dex had a significant effect on the activity of TACE but not on its expression level in activated RAW cells.

To further address how Dex regulates the activity of TACE, we hypothesized that Dex may be a potent TACE inhibitor. Using an *in vitro* TACE inhibitor assay kit (TACE inhibitor screening assay kit), we showed that Dex did not inhibit the activity of purified TACE proteins (Figure 3D), which implies that Dex indirectly brings about the suppression of LPS-induced TACE activation. Thus, we further investigated the effect of Dex on LPS-induced p38 MAPK activation, a kinase that enhances TACE activation via the direct phosphorylating TACE at Thr735 [18]. We found that the induced p38 phosphorylation at Thr180/Tyr182 by LPS was significantly suppressed soon after 30 min of Dex treatment (Figure 3E and Supplementary Figure 2), although the induced level of phospho-ERK1/2 (Thr202/Tyr204) and phospho-JNK (Thr183/Tyr185) were not changed by Dex treatment (Supplementary Figure 2). In addition, because Toll-like receptor 4 has been reported to be a LPS receptor that mediates the LPS-induced inflammatory response [39], we also examined whether Dex has an effect on LPS-induced Toll-like receptor signaling. We found that transforming growth factor-β-activated kinase 1 (TAK1) and IκB kinase-α/β (IKK-α/β) were activated by phosphorylation soon after 30 min of LPS stimulation, and became inactive again before Dex treatment in our study system (Figure 3E). Thus, in our model system, Dex appears to act on LPS-induced p38α MAPK signaling, which then results in TACE inactivation and a retardation of TNF-α secretion.

### The hydroxyl groups of dexamethasone serve as the functional groups that control LPS-induced TNF-α secretion

**Figure 4:**
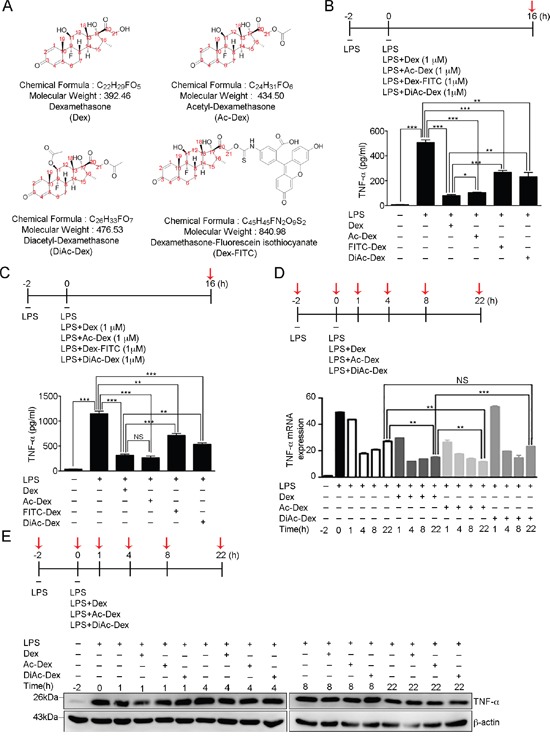
The 11, 21-hydroxyl groups of dexamethasone serve as the functional groups in controlling LPS-induced TNF-α secretion **(A)** The chemical structures, formulae and molecular weights of dexamethasone (Dex), acetyl-dexamethasone (Ac-Dex), diacetyl-dexamethasone (DiAc-Dex), and dexamethasone-fluorescein isothiocyanate (Dex-FITC). **(B)** The concentration of secreted TNF-α in the medium of RAW264.7 cell culture was determined after the indicated treatments at the time point highlighted with red arrow. The graph represents the mean ± s.e.m. (n = 3 independent experiments). *p <0.05; **p <0.01; ***p < 0.001. **(C)** The concentration of secreted TNF-α was determined in the medium of BMDMs cell culture after the indicated treatments at the time point highlighted with red arrow. The graph represents the mean ± s.e.m. (n = 3 independent experiments). **p <0.01; ***p < 0.001; NS, no significance. **(D)** Fold change in TNF-α mRNA expression was determined for RAW264.7 cells after the indicated treatments at the six time points highlighted with red arrows relative to that at the first time point (−2 h). The graph represents the mean ± s.e.m. (n = 3 independent experiments). **p <0.01; ***p < 0.001; NS, no significance. **(E)** Cell lysates from RAW264.7 cells after the indicated treatments at the time points highlighted with red arrows were analyzed by Western blotting using antibodies against TNF-α and GAPDH.

Within the chemical structure of Dex (Figure 4A), we proposed that the molecule's hydroxyl groups potentially are the functional groups that mediate anti-inflammation. Previously, we have found that dexamethasone-fluorescein isothiocyanate (Dex-FITC), which is formed by conjugating fluorescein isothiocyanate (FITC) to Dex at the molecule's 21-hydroxyl group (Figure 4A) was unable to enter the cell (Supplementary Figure 3), which implies an important role for the 21-hydroxyl group of Dex. In order to further examine the functionality of the 11-hydroxyl and 21-hydroxyl groups of Dex in terms of anti-inflammatory function, we generated acetyl-dexamethasone (Ac-Dex) and diacetyl-dexamethasone (DiAc-Dex) by acetylating either the 21-hydroxyl group of Dex only or both the 11-hydroxyl and 21 hydroxyl groups of Dex, respectively (Figure 4A). Next, the level of soluble TNF-α in culture medium of activated RAW cells and BMDMs was analyzed using the various indicated treatment (Figure 4B and 4C). The results indicated a suppression of LPS-induced soluble TNF-α secretion by Dex, but both of FITC-Dex and DiAc-Dex showed suppressed anti-inflammatory effect (Figure 4B and 4C). However, surprisingly, modification at the 21-hydroxyl group only with an acetyl group (Ac-Dex) resulted in a molecule that still retained an anti-inflammatory effect. One possibility is that the size differences between acetyl group on Ac-Dex and FITC on FITC-Dex may be responsible. However, the results clearly show that modification at both the 11-hydroxyl and 21-hydroxyl groups (DiAc-Dex) resulted in a significant disruption of the effect of Dex and produced a dramatically higher level of soluble TNF-α in culture medium than Dex treatment (Figure 4B and 4C). It seems likely that the 11-hydroxyl group and 21-hydroxyl group are directly involved in the anti-inflammatory activity of Dex, although the possibility remains that DiAc-Dex may not be able to enter the cell, just like FITC-Dex (Supplementary Figure 3), although give the very small size differences between Dex, Ac-Dex and DiAc-Dex, this seems unlikely. Alternatively, both hydroxyl groups are required for uptake into the cell.

We next examined the level of TNF-α mRNA production induced by LPS in activated RAW cells with the various indicated treatment (Figure 4D). Real-time q-PCR previously had revealed a two-phase peak for TNF-α mRNA induction by LPS (Figure 4D), as the results show in Figure 1C. Treatment with Dex, Ac-Dex or DiAc-Dex did not suppress the initial induction of LPS-induced TNF-α mRNA. However, the second phase of TNF-α mRNA production was only detected in activated RAW cells treated with LPS+DiAc-Dex, but not in cells treated with LPS+Dex or LPS+Ac-Dex (Figure 4D), which supports the results in Figure 4B and 4C, namely that DiAc-Dex has lost ability to suppress TNF-α secretion via the autocrine activation of cell surface TNF-α receptor signaling that induce the second phase of TNF-α production [35, 36]. Furthermore, we also confirmed that the amount of TNF-α protein induced by LPS was not suppressed by Dex, Ac-Dex or DiAc-Dex treatment up to the 22-hour time point (Figure 4E).

**Figure 5:**
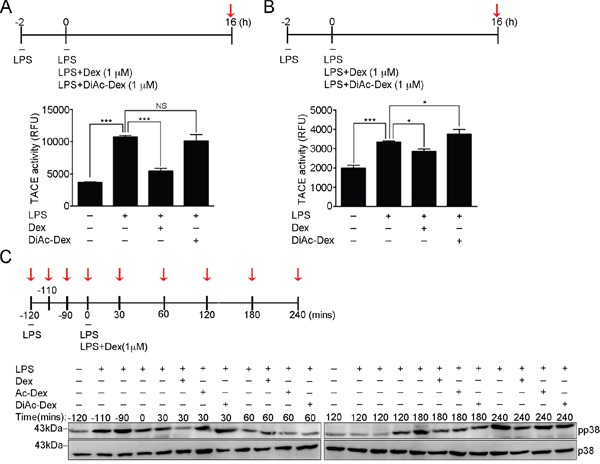
The 11, 21-hydroxyl groups of dexamethasone serve as the functional groups and control LPS-induced p38-mediated TACE activation **(A)** The proteolytic activity of TACE from RAW264.7 cells after the indicated treatments at the time point highlighted with red arrow. The graph represents the mean ± s.e.m. (n = 3 independent experiments). **(B)** The proteolytic activity of TACE from BMDMs cells after the indicated treatments at the time point highlighted with red arrow. The graph represents the mean ± s.e.m. (n = 3 independent experiments). **(C)** Cell lysates from RAW264.7 cells after the indicated treatments at the time points highlighted with red arrows were analyzed by Western blotting using antibodies against p38 MAPK and p-p38 [phospho-p38 MAPK (Thr180/Tyr182)].

To assess the contribution of the hydroxyl groups of Dex to the attenuation of LPS-induced TACE activation, we measured cellular TACE activity in activated RAW cells (Figure 5A) and BMDMs (Figure 5B) that had been continuously treated with LPS only, LPS accompanied by Dex or LPS accompanied by DiAc-Dex for 16 hours. DiAc-Dex was unable to suppress LPS-induced TACE activation compare to LPS alone, while Dex did significantly suppressed LPS-induced TACE activation (Figure 5A and 5B). Furthermore, we further examined the effect of DiAc-Dex on LPS-induced p38 phosphorylation, and found that the phosphorylation level of p38 was significantly suppressed after 30 min of Dex treatment (Figure 5C), as shown in Figure 3E and Supplementary Figure 2, but this did not occur with DiAc-Dex (Figure 5C). These findings suggest that Dex via its hydroxyl groups appears to act on LPS-induced p38α MAPK signaling to suppress TACE activation, which in turn leads to the suppression of LPS-induced TNF-α secretion.

### Acetylated dexamethasone cannot rescue mice from LPS-induced lethality

**Figure 6:**
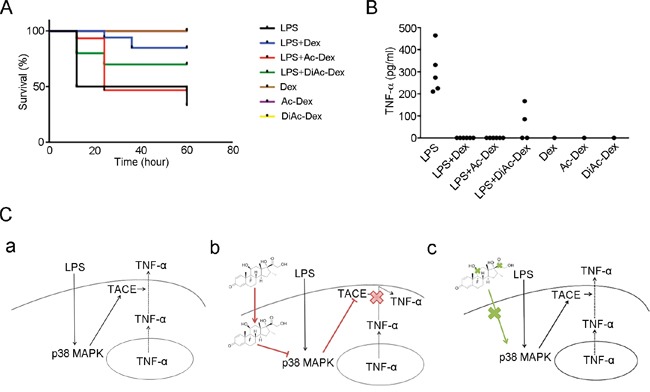
Dexamethasone, via the molecule's hydroxyl groups, controls LPS-induced lethality and anti-inflammatory responses **(A)** Kaplan-Meier plot of mice treated with LPS (50 mg/kg) only (n=9), LPS (50 mg/kg) + Dex (10 mg/kg) (n=7), LPS (50 mg/kg) + Ac-Dex (10 mg/kg) (n=7), LPS (50 mg/kg) + DiAc-Dex (10 mg/kg) (n=7), Dex (10 mg/kg) only (n=1), Ac-Dex (10 mg/kg) (n=1) or DiAcDex (10 mg/kg) (n=1). *p* <0.01 for LPS + Dex versus LPS only; NS for LPS + Ac-Dex versus LPS only and LPS + DiAc-Dex versus LPS only. **(B)** The concentration of TNF-α in the serum of mice 24 h after the indicated treatments. Each data point in the graph represents one individually analyzed mouse. **(C)** Model of how Dex acts as an immunosuppressant. **(a)** Initially LPS activates the p38 MAPK pathway and this is followed by TACE activation, which controls TNF-α secretion. **(b)** In activated macrophages, treatment with Dex suppresses LPS-induced TACE activation by inhibiting p38 MAPK pathway, thereby attenuating the induction of TNF-α secretion. **(c)** Dex without functional hydroxyl groups is not able to attenuation of LPS-induced TNF-α secretion.

To assess the relative importance of the hydroxyl groups of Dex *in vivo*, we treated mice with LPS and accompanied this with vehicle, Dex, Ac-Dex or DiAc-Dex. A mortality of 77.8% was noted among the vehicles-treated mice, whereas the mortality of the Dex-treated mice was reduced to 28.6% (Figure 6A). In agreement with the improved survival of the Dex treated mice, we observed only 71.4% or 57.1% mortality among mice treated with LPS when this was accompanied by Ac-Dex or DiAc-Dex, respectively (Figure 6A), while mice treated with Dex, Ac-Dex or DiAc-Dex alone showed 0% mortality. We also measured the level of TNF-α in the serum of mice treated with LPS accompanied by vehicles or DiAc-Dex (Figure 6B). Together, these findings confirm the importance of the hydroxyl groups of Dex in the suppression of LPS-mediated inflammatory signaling through the down-regulation of the p38 MAPK pathways and TACE activation, which then leads to a retardation of TNF-α secretion and reduced mortality in mice.

## DISCUSSION

Our study has uncovered the functional groups on Dex that act to oppose innate immune reactions and has clarified the mechanism by which Dex suppresses TNF-α release. We have focused on the effect of Dex on the release of the pro-inflammatroy cytokine TNF-α. We have shown that Dex controls TNF-α secretion, but does not affect gene expression of TNF-α and that it acts via p38 MAPK-mediated TACE activation. LPS-induced activation of p38 MAPK is inhibited by Dex, thereby blocking the TACE activation needed for TNF-α shedding and secretion. When both the 11-hydroxyl and the 21-hydroxyl groups of Dex are blocked, Dex fails to show an immunosuppresant effect. The effect of Dex on the innate immune reactions induced by LPS would seem to be via the molecule's hydroxyl groups and these also seem to be critical to the control of p38 MAPK signaling, TACE activity and pro-inflammatory cytokine TNF-α secretion (Figure 6C).

Our results have revealed for the first time that the functional groups of Dex are involved in regulating the innate immune responses induced by bacterial LPS, although Dex has been previously documented as having pleiotropic effects regarding inhibition of cytokine release [32, 33]. By assessing the effect of Dex using mice, we have shown that Dex protects mice from not only pulmonary inflammation, but also mortality due to treatment with LPS (Figure 6A), which supports the notion that Dex acts in a similar manner to glucocorticoid hormones, namely that it can control innate immune responses [40]. Glucocorticoids pass through cell membrane and bind to glucocorticoid receptors in the cytoplasm, from where they are carried by the dynein motor to the nucleus [41]. There they bind to the glucocorticoid response elements that control the transcriptional regulation of cytokines [30]. It seems likely that Dex may exert its effects via the same mechanism. One important possibility is that the hydroxyl groups of Dex may play a crucial role in the passing of Dex through the cell membrane in order to exert its anti-inflammatory ability. Our findings clearly show that when there is a dual blockage of the hydroxyl groups of Dex, the molecule is unable to efficiently suppress the secretion of TNF-α by activated macrophage (Figure 4B and 4C). In addition, the blockage of both hydroxyl groups abolished the ability of Dex to protect mice from LPS-induced death (Figure 6A). Interestingly, while at an *in vitro* level there seems to be little difference between Ac-Dex and Dex, at an *in vivo* level Dex treatment results in mortality of only 28.6%, but this is increased to 71.4% when Ac-Dex is used. This should be compared to 57.1% for DiAc-Dex and 77.8% for vehicle. Thus it would seem that the hydroxyl groups present on Dex play an essential part in the molecule's anti-inflammatory ability.

Our findings, together with previous studies, support the notion that the secretion of the cytokine TNF-α, when induced by LPS, is abolished by the synthetic glucocorticoid Dex [33]. Indeed, we have shown that Dex treatment results in changes to p38 MAPK signaling, which leads to TACE inactivation and the suppression of TNF-α shedding which is needed for TNF-α secretion. TNF-α has been characterized as a pro-inflammatory cytokine that is overexpressed at both the transcriptional and translational level upon LPS stimulation; this is suppressed by pretreatment with Dex [32]. Nevertheless, this study has shown that, in activated macrophages stimulated with LPS for 2h, Dex does not reduce the initial induced level of TNF-α mRNA (Figure 1C), indicating that Dex did not exert its effect at the transcriptional level at this early stage of activated macrophages. Our findings also have revealed that, upon Dex treatment, the second phase of TNF-α mRNA production that is induced by LPS is abolished (Figure 1C), which implies that autocrine TNF-α signaling [35, 36] is inhibited by Dex treatment due to the suppression of soluble TNF-α secretion. To date, aberrant TNF-α production is believed to be involved in TNF receptor signaling via two transmembrane receptors, these are TNF receptor 1 (TNFR1) and TNF receptor 2 (TNFR2). These receptors have been associated with the pathogenesis of several inflammatory diseases [35]. As Dex is able to exert its act at the level of TNF-α secretion, the suppression of soluble TNF-α production by activated macrophages would seem to involve inhibition of TNF receptor signaling and a blocking of the inflammatory cytokine cascades.

Several critical questions remain about Dex-mediated inactivation of TACE signaling. TACE has been shown to be activated via p38 MAPK signaling [18], the activity of which can be regulated either by MKK3/MKK6 among the Toll-like receptor signals [1] or by mitogen-activated protein (MAP) kinase phosphatase-1 (MKP-1) among the glucocorticoid receptor signals [31]. By examining the effect of Dex on LPS-induced Toll-like receptor signaling, the downstream molecules, TAK1 and IKK-α/β, are activated soon after 30 min of LPS stimulation and turned inactive again before Dex treatment in our study system (Figure 3E). While the phosphorylation level of p38 at Thr180/Tyr182, which is induced by LPS, is suppressed by Dex treatment, the molecules upstream of p38 signaling in the glucocorticoid receptor signaling pathways remain a possible target for Dex. Thus there remains the possibility that Dex-mediated TACE inactivation may occur via other signaling molecules upstream of p38 MAPK and this needs to be explored. Additionally, the mechanism(s) by which the structure of Dex exerts control on signaling molecules and regulates the signaling pathways remain unknown. Future studies are clearly needed to help clarify these important questions.

## MATERIALS AND METHODS

### Cells and reagents

Murine monocytes/macrophages (RAW264.7) were provided by Prof. Ping-Hui Tseng's laboratory and were maintained in DMEM-high glucose (Invitrogen) supplemented with 10% heat inactivated FBS (Invitrogen) and 1% antibiotics solution (penicillin and streptomycin; Invitrogen) under 5% CO_2_. Bone Marrow-derived Macrophage (BMDMs) were differentiated from the bone marrow cells collected from 6-8 weeks old mice using L-cell conditional medium [42].

Lipopolysaccharide (LPS; working concentration: 0.1μg/ml) and dexamethasone (Dex) were purchased from Sigma. Dexamethasone-fluorescein (Dex-FITC) was purchased from Thermo Fisher. RU486 was purchased from Merck. Ac-Dex and DiAc-Dex were produced via a series of chemical reactions by Prof. Chung-Wai Shiau's laboratory.

### Animal handling

The mouse protocols were approved by the Institutional Animal Care and Use Committee (IACUC) of National Yang-Ming University. The mice were housed on a 12-hour light and 12-hour dark cycle. The mice used for the experiments were 8 to 10 weeks old and were of C57BL/6 male.

### Antibodies

The sources of the antibodies and their dilutions were as follows: rabbit anti-TNF-α (Merck Millipore AB2148P; dilution for Western blot: 1/1000; dilution for immunofluorescence: 1/200); mouse anti-Rab11 (BD 610656; dilution for immunofluorescence: 1/200); rabbit anti-MAPK (ERK1/2) (Santa Cruz sc-154; dilution for Western blotting: 1/1000); rabbit anti-phospho-p44/42 MAPK (ERK1/2) (Thr202/Tyr204) (Cell Signaling #4370s; dilution for Western blotting: 1/1000); mouse anti-JNK (Santa Cruz sc-7345; dilution for Western blotting: 1/1000); rabbit anti-phospho-JNK (Thr183/Tyr185) (Cell Signaling #9251s; dilution for Western blotting: 1/1000); rabbit anti-p38 MAPK (Cell Signaling #9212; dilution for Western blotting: 1/1000); rabbit anti-phospho-p38 MAPK (Thr180/Tyr182) (Cell Signaling #9211; dilution for Western blotting: 1/1000); rabbit anti-TAK1 (Santa Cruz sc-7162; dilution for Western blotting: 1/2000); rabbit anti-phospho-TAK1 (Thr184/187) (Cell Signaling #4531; dilution for Western blotting: 1/1000); rabbit anti-IKK-α/β (Santa Cruz sc-7607; dilution for Western blotting: 1/2000); rabbit anti-phospho-IKK-α (Ser176)/IKK-β (Ser177) (Cell Signaling #2078; dilution for Western blotting: 1/1000); mouse anti-β-actin (GeneTex GTX629630); dilution for Western blotting: 1/1000); mouse anti-GAPDH (Santa Cruz sc-137179; dilution for Western blotting: 1/1000); mouse anti-TNF receptor I (CD120a) (eBioscience #16-1202-81; dilution for flow cytometry: 1/200); goat anti-TACE (ADAM17) (Santa Cruz sc-6416; dilution for Flow cytometry: 1/50); Alexa Fluor 488-anti-mouse IgG (Invitrogen A11029; dilution for immunofluorescence: 1/300; dilution for flow cytometry: 1/300); Alexa Fluor 488-anti-rabbit IgG (Invitrogen A11034; dilution for immunofluorescence: 1/300); Alexa Fluor 568-anti-rabbit IgG (Invitrogen A11036; dilution for immunofluorescence: 1/300); Cy5-anti-goat IgG (Jackson ImmunoResearch 705-175-147; dilution for flow cytometry: 1/300); HRP-AffiniPure mouse anti-rabbit IgG (Jackson ImmunoResearch 211-032-171); and HRP-AffiniPure goat anti-mouse IgG (Jackson ImmunoResearch 115-035-174).

### RNA extraction, reverse transcription and real-time quantitative PCR

RNA was extracted from cells using TRIzol reagent (Invitrogen) and total RNA was precipitated according to the manufacturer's instructions. The RNA products were reverse-transcribed using Transcriptor First Strand cDNA Synthesis kits (Roche) and random hexamer primers. The cDNA products were amplified by PCR using a KAPA SYBR® FAST ABI Prism™ One-Step qRT-PCR kits (KAPA BIOSYSTEMS). The CPH gene was used as a reference gene. Quantification of the target mRNA was carried out by the ΔΔCT method.

The qPCR primers for TNF-α were 5′-CGTAGGCGATTACAGTCACGG-3′ and 5′-GACCAG GCTGTCGCTACATCA-3′. The qPCR primers for TACE were 5′-TGTGAGCGGTGACCACGAGAAT-3′ and 5′-TTCATCCACCCTGGAGTTGCCA-3′. The qPCR primers for CPH were 5′-ATGGTCAACCCCACCGTGT-3′ and 5′-TTCTTGCTGTCTTTGGAACTTTGTC-3′.

### Mouse TNF-α ELISA

To analyze the level of TNF-α secretion, samples of medium and serum were assayed for TNF-α content using Mouse TNF-α DuoSet ELISA kits (R&D systems) according to the manufacturer's instructions. The results are presented graphically using Excel software (Microsoft).

### Immunofluorescence microscopy

Cells were fixed and immunostained using a method that has been described previously [43]. Briefly, cells were fixed with 4% paraformaldehyde at room temperature for 20 min, permeabilized with PBS containing 0.01% Triton X-100 and 0.05% SDS at room temperature for 5 min, and then blocked with blocking solution (0.1% saponin and 0.2% BSA in PBS) for 30 min. Subsequently, the cells were incubated with the indicated primary antibodies in blocking solution overnight at 4°C, and then incubated with fluorescent dye–conjugated secondary antibody 1 h. Cells were mounted on slides with fluorescent mounting medium (Dako). Confocal images were acquired using a 100X 1.49NA (Oil-Immersion) Plan objective lens (Nikon) and a 1.5X magnification lens attached to a Nikon Ti-E equipped with *iLas* multi-modal of TIRF (Roper)/spinning disk confocal (CSUX1, Yokogawa) microscope system and a ProEMCCD camera (Princeton). All confocal images were captured and processed using Metamorph software (Molecular Device). To provide orthogonal projections of a given cell, Metamorph image analysis software was used.

### Cell lysates and Western blotting

Cells were washed twice with ice-cold PBS and lysed with RIPA solution (50 mM Tris-HCl pH8.0, 2 mM EDTA, 150 mM NaCl, 1% NP40, 0.1 % SDS, 0.5 % sodium deoxycholate) supplemented with protease inhibitor cocktails and phosphatase inhibitor cocktails (Roche) at 4°C for 20 min. The cell lysates were then sonicated, which was followed by centrifugation in order to collect the supernatants for protein concentration quantification by Bradford protein assay (Bio-Rad). Equal amounts of total protein per sample was run on SDS-PAGE (SDS-polyacrylamide gel electrophoresis) and then transferred to PVDF membrane (Millipore). The blots were probed using the indicated primary antibodies. Visualization was carried out using mouse anti-rabbit horse radish peroxidase-conjugated secondary antibody (HRP-AffiniPure mouse anti-rabbit IgG) or goat anti-mouse horse radish peroxidase-conjugated secondary antibody (HRP-AffiniPure goat anti-mouse IgG) as appropriate follow by Immobilon Western Chemiluminescent HRP substrate (Millipore). The results were captured using a Luminescence Image System (FUJIFILM).

### Flow cytometry

To assess the cell surface expression of TNF receptor I (CD120a) and TACE (ADAM17), cells were immunostained using a method that has been described previously [43]. Briefly, cells were washed with ice-cold PBS and re-suspended in blocking solution (PBS containing 1% BSA). The cells were then incubated with anti-TNF receptor I or anti-TACE antibodies for 1 h on ice, washed with blocking solution, and labeled with Alexa Fluor 488-anti-mouse IgG (for TNF receptor I) or Cy5-anti-goat IgG (for TACE) for 30 min on ice. Next the cells were washed, which was followed by analysis on a BD LSRFORTESSA (BD Bioscience). The datasets collected were analyzed using FlowJo software.

### Cellular TACE activity assay

To analyze TACE activity, cell lysate samples were assayed for TACE activity using SensoLyte® 520 TACE Activity Assay kits (Anaspec) according to the manufacturer's instructions. The results are presented graphically using Excel software (Microsoft).

### TACE inhibitor screening assay

To examine the inhibitory effects of dexamethasone on TACE activity, TACE inhibitor screening assay kits assays (BioVision) were performed according to the manufacturer's instructions. The results are presented graphically using Excel software (Microsoft).

### Statistical analysis

Statistical significance was measured by Student's t-test.

## SUPPLEMENTARY FIGURES



## Abbreviations

Dex: dexamethasone
LPS: Lipopolysaccharide
TNF-α: Tumor necrosis factor-α
TACE: Tumor necrosis factor-α (TNF-α)-converting enzyme
