# Supplementary material for: Suppression of LPS-induced inflammatory responses by the hydroxyl groups of dexamethasone
Source: Oncotarget. 2017 May 8;8(30):49735–48. doi: 10.18632/oncotarget.17683 (PMC5564803; doi:10.18632/oncotarget.17683)
Supplement: Supplementary file 1 [file oncotarget-08-49735-s001.pdf]

Suppression of LPS-induced inflammatory responses by the hydroxyl groups of dexamethasone

SUPPLEMENTARY MATERIALS

SUPPLEMENTARY FIGURES

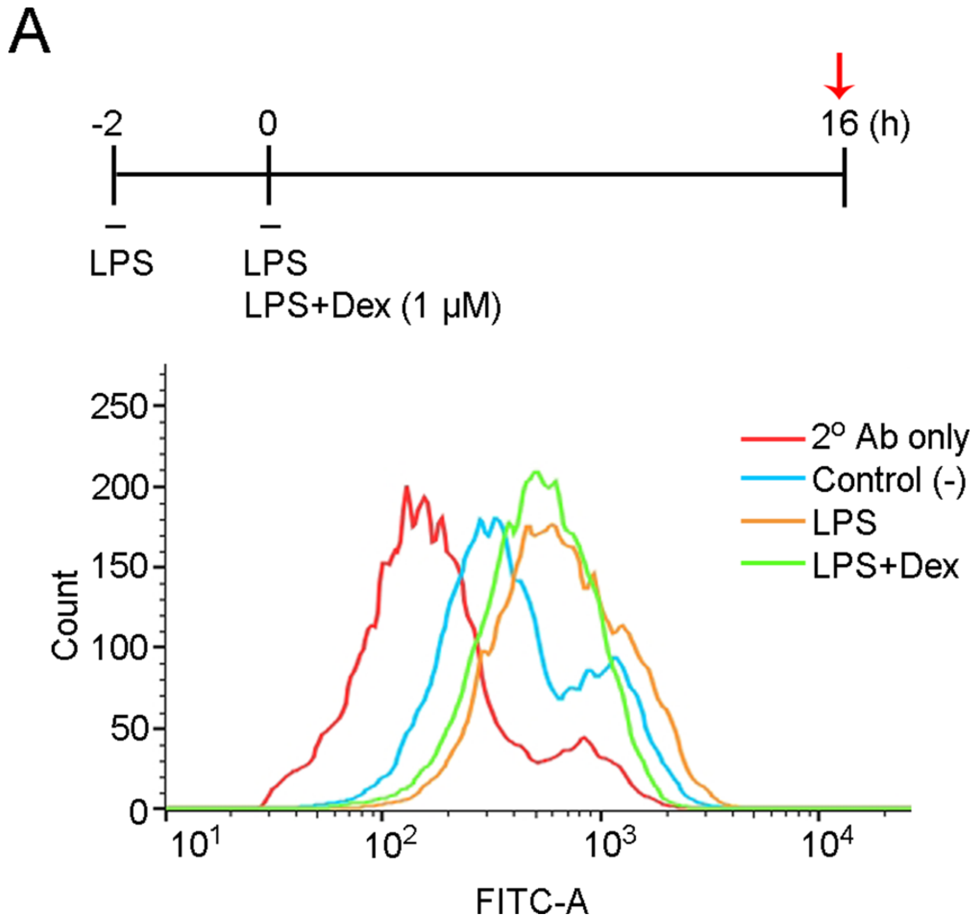

Supplementary Figure 1: Dexamethasone does not influence the expression of TNF receptor 1 on the cell surface. The effect of Dex on the cell surface expression of TNF receptor 1. RAW264.7 cells without (control) or with the indicated treatments were incubated with TNF receptor 1 antibody or buffer alone (2nd Ab only), followed by labeling with Alexa Fluor 488-conjugated secondary antibody. Fluorescence intensity was detected using flow cytometry and determined using FlowJo software. The results of a representative experiment from three independent experiments are shown.

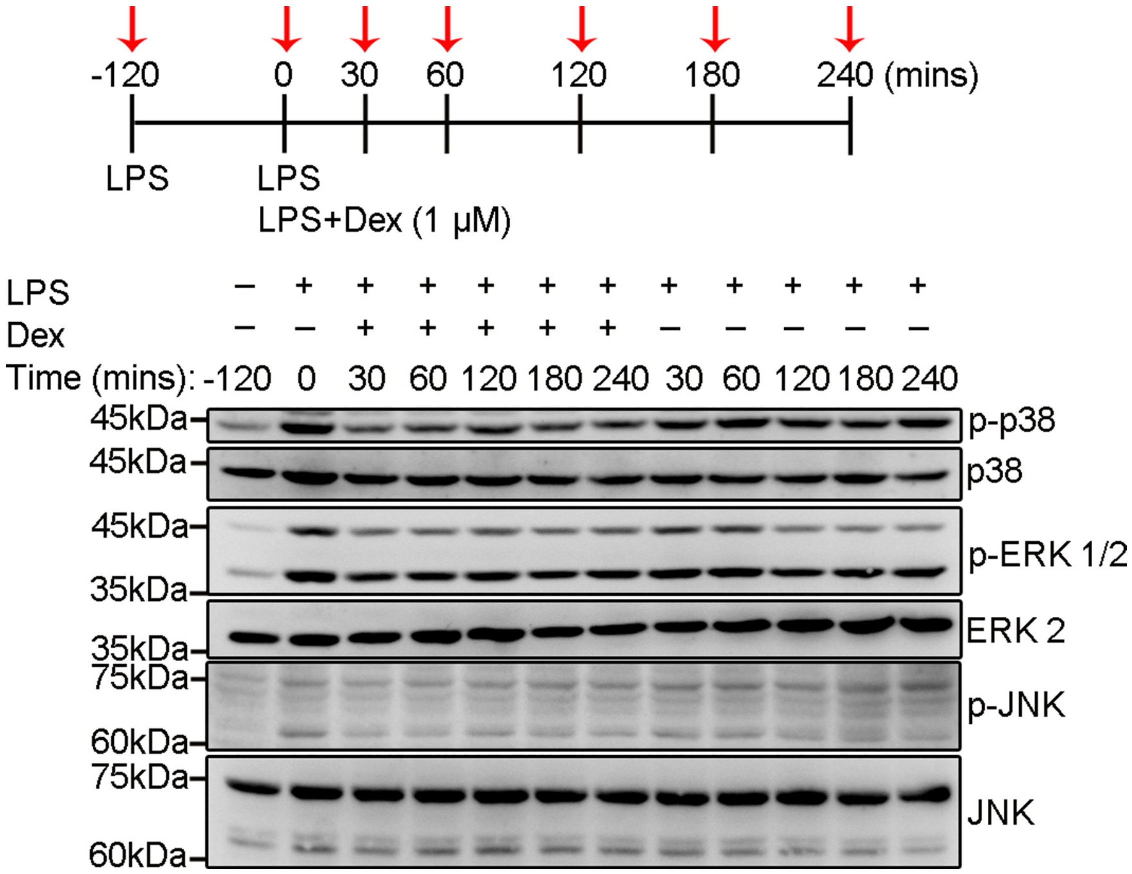

Supplementary Figure 2: Dexamethasone inactivates p38MAPK signaling. Cell lysates from RAW264.7 cells after the indicated treatments at the seven time points highlighted with red arrows were analyzed by Western blotting using antibodies against ERK2, p-ERK1/2 [phospho-p44/42 ERK1/2 (Thr202/Tyr204)], p38 MAPK, p-p38 [phospho-p38 MAPK (Thr180/Tyr182)], JNK and p-JNK [phospho-JNK (Thr183/Tyr185)].

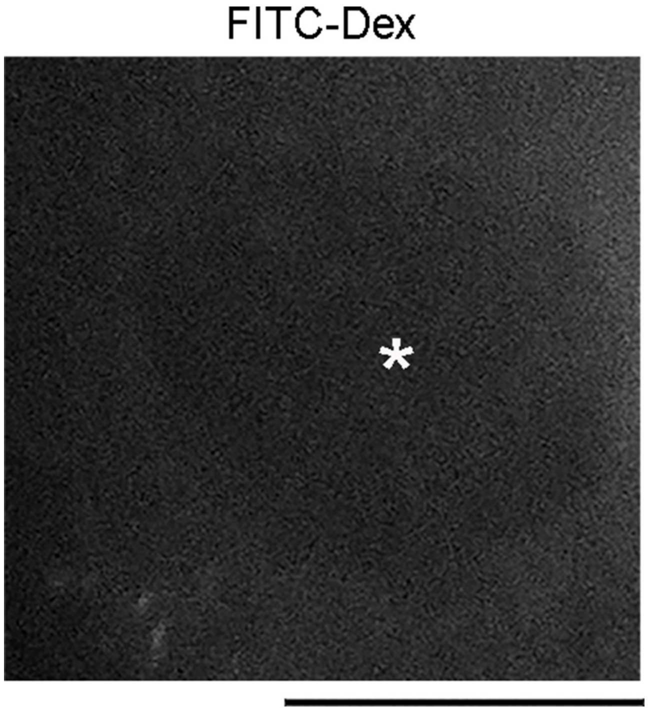

Supplementary Figure 3: FITC-Dexamethasone loses the ability to permeable cell membrane. Confocal images of RAW264.7 cells treated with FITC-Dex. The star (*) indicates the location of cell. FITC-Dex was distributed in the medium but not inside of a cell. Bar, 10 µm.
